# Regulation, resources, and job burnout among traffic auxiliary police in China: evidence from G City

**DOI:** 10.3389/fpubh.2026.1855841

**Published:** 2026-08-04

**Authors:** Fanghui Zheng, Chengcheng Liu, Yu Wen, Jiongshan Zou

**Affiliations:** 1 School of Public Administration, South China University of Technology, Guangzhou, China; 2 Guangxi University, Nanning, China; 3 School of Public Administration, Xiangtan University, Xiangtan, China; 4 International School, Jinan University, Guangzhou, China

**Keywords:** digital resources, job burnout, job demands-resources model, job satisfaction, regulation, traffic auxiliary police

## Abstract

**Background:**

Traffic auxiliary police often work under stringent institutional regulation while receiving limited organizational and digital support, a combination that may heighten the risk of burnout. Evidence on how regulatory demands and work-related resources jointly shape burnout in this occupational group remains limited.

**Methods:**

Using a multistage stratified sample of 502 traffic auxiliary police officers in G City, southern China, this study integrates regulatory theory with the job demands-resources (JD-R) model to examine the associations of job regulation demands and job resources with burnout. Multiple linear regression, mediation analysis, bootstrap testing, and moderation analysis were used to assess the roles of job satisfaction and digital resources.

**Results:**

Job regulation demands were positively associated with burnout, whereas job resources were negatively associated with burnout. Among the three burnout dimensions, emotional exhaustion showed the strongest association. Regulatory pressure constraints and regulatory environment discrepancies showed relatively consistent positive associations with burnout, whereas regulatory role tension was associated with selected burnout dimensions. Among the job-resource dimensions, organizational resource support showed the most consistent negative association with burnout, while career performance feedback and work autonomy resources showed more dimension-specific or weaker associations. Job satisfaction partially mediated these relationships, and digital resources moderated them.

**Conclusion:**

Burnout among grassroots traffic auxiliary police reflects a structural imbalance between regulatory demands and available resources. Reducing excessive regulatory pressure, strengthening institutional and digital support, and adopting satisfaction-oriented management may help protect occupational health in grassroots collaborative law enforcement.

## 1 Introduction

Public health has long been closely linked to regulation, and appropriately designed regulatory arrangements can exert both enabling and constraining effects on public health governance (1). Regulation through legal rules, institutional requirements, and resource allocation lies at the core of regulatory theory. In practice, regulatory systems rely on coercive and organizational instruments to guide or constrain the behavior of individuals and institutions in order to maintain social order with limited direct intervention (2). When regulation becomes excessively intensive or poorly aligned with available resources, however, it may deepen perceived inequality among frontline personnel and undermine their psychological well-being. Traffic auxiliary police are an important group within grassroots collaborative law enforcement. Under pressure-driven governance, they face not only routine procedural control but also red tape associated with campaign-style enforcement and digital transformation (3), both of which may reduce job satisfaction and increase burnout risk. In this context, burnout-related problems among auxiliary police, including occupational health inequality and growing psychological strain, have increasingly become a public-health concern and may also have downstream implications for policing practice, public safety governance, law-enforcement quality, and road safety (4–6).

Both auxiliary police and regular police face elevated public-health and psychosocial risks (6). Auxiliary police differ from regular police in both institutional status and access to work-related resources (7–9). Historically, auxiliary policing can be traced to arrangements such as the British “Special Constable,” which emphasized voluntary support roles (10). In China, auxiliary police are generally understood as non-established personnel who assist public security organs in law enforcement, crime prevention, social order maintenance, and public service delivery (11), including duty-based, clerical, and technical positions (12). Compared with formally established police officers, auxiliary police generally face lower entry thresholds, less stable career prospects, fewer opportunities for promotion, and weaker access to autonomy, psychological support, and professional development resources. Most serve in repetitive, physically demanding, and high-intensity frontline roles, often with substantially lower pay and welfare benefits than regular police officers (13). Because their salaries are financed through public budgets, auxiliary police can also be regarded as fiscally supported public-sector workers. At the same time, the absence of formal staffing places them in a semi-formal institutional position that is neither fully external to the state nor fully incorporated into the regular establishment (14).

Traffic auxiliary police currently work under strict regulatory constraints and limited resource support, conditions that make burnout and mental health inequality particularly salient (11). They constitute the main auxiliary workforce on the frontline of traffic management and are responsible for traffic guidance, accident scene maintenance, security duties, order stabilization, and the persuasion of violators. At the same time, road safety remains a major public-health challenge. WHO estimates suggest that approximately 1.19–1.3 million people die in road traffic accidents worldwide each year, and the China Statistical Yearbook 2025 reports 260,072 road traffic accidents in China in 2024, with 59,280 deaths and direct property losses of 1,349.053 million yuan (15). In many settings, the number of auxiliary police now exceeds that of regular police officers (7). Yet continuing debates over the legal status, authority boundaries, and institutional identity of auxiliary police have weakened the legitimacy of their participation in partially independent policing tasks (13). Ambiguous authorization, an imbalance between responsibilities and powers, and fragmented job resources further undermine their work well-being. Understanding how to balance regulatory pressure and resource support is therefore important both for reducing burnout and for improving the standardized governance of grassroots fiscally supported personnel.

A substantial body of public mental health research has examined the concept, measurement, antecedents, and consequences of job burnout. Burnout is commonly defined as a state of physical and psychological exhaustion accompanied by negativity or detachment that develops under prolonged job stress (16); it is closely linked to core aspects of work engagement, such as energy, commitment, and effectiveness. Once established, burnout depletes emotional, cognitive, and physical resources and can adversely affect work attitudes, mental and physical health, and performance outcomes.

In the context of rule-of-law governance in the new era, characterized by institutionalized co-governance and the reconfiguration of governance institutions, a regulatory perspective can be understood as an institutional lens for analyzing burnout-related job demands. Rather than conceptualizing demands merely as workload, emotional labor, or generalized time pressure, this perspective focuses on pressures generated by formal and informal rules, hierarchical supervision, accountability requirements, and heterogeneous enforcement environments. For traffic auxiliary police, such system-based demands are especially salient because they work in semi-formal positions under high-intensity rule enforcement, limited staffing security, and contested authority boundaries. This perspective therefore extends the demand side of the JD-R model from generic work strain to institutionally embedded regulatory demands that frequently arise in grassroots collaborative law-enforcement settings (2, 3, 17–21).

Accordingly, this study addresses one overarching research question: how do regulation and resources influence job burnout among grassroots traffic auxiliary police in China? This question is further divided into three sub-questions: (1) How can the JD-R model be adapted from a regulatory perspective to analyze job burnout? (2) Which regulatory demands and job resources are most strongly associated with burnout among grassroots traffic auxiliary police, and through what pathways do they operate? (3) How might burnout in this group be reduced more effectively? In line with these questions, the study pursues three objectives: to develop a regulatory-oriented JD-R analytical framework; to examine the relationships among job regulation demands, job resources, job satisfaction, digital resources, and burnout; and to provide empirical evidence for reducing burnout and improving occupational mental health among grassroots governance workers.

To answer these questions, we integrate regulatory theory with the JD-R model to construct a “regulation–resources” analytical framework and test it using survey data from G City, southern China. Based on 502 valid questionnaires, we employ multiple linear regression, mediation analysis, and moderation analysis to examine the relationships among job regulation demands, job resources, job burnout, job satisfaction, and digital resources. The focus on traffic auxiliary police is justified for three reasons. First, this group provides a useful empirical setting for assessing the external validity of burnout theory in a highly institutionalized context that relies heavily on non-established personnel. Second, grassroots governance in China depends substantially on fiscally supported off-establishment workers, making this group important for understanding broader patterns of occupational inequality. Third, traffic auxiliary police constitute a common category of non-established public employees, and their burnout has direct implications for standardized management, collaborative law enforcement, and human resource allocation at the grassroots level.

This study contributes to the literature at both the theoretical and practical levels. Theoretically, it brings regulatory theory into dialogue with the JD-R literature by conceptualizing job regulation demands and institutionally embedded job resources as key antecedents of burnout. It also broadens existing explanations of burnout by examining job satisfaction as a mediating mechanism and digital resources as a moderating condition. Empirically, it extends burnout research beyond formally established staff to a large but underexamined group of semi-formal public-sector workers. In doing so, it provides new evidence for understanding the staffing paradox of “in-staff compression” alongside “off-staff expansion” and for informing talent governance at the grassroots level.

The remainder of this article is organized as follows. Section 2 develops the theoretical framework and hypotheses. Section 3 describes the data source, measurement strategy, model specification, and statistical procedures. Section 4 presents the empirical results. Section 5 interprets the findings, discusses their theoretical and practical implications, outlines the study limitations, and concludes.

## 2 Theory and hypotheses

### 2.1 Theoretical framework

Research on job burnout has generated extensive discussion of its definition, determinants, and theoretical underpinnings. The World Health Organization defines burnout as a syndrome resulting from chronic workplace stress that has not been successfully managed, characterized by energy depletion, increased mental distance or cynicism toward one’s job, and reduced professional efficacy (22). Maslach’s classic framework likewise conceptualizes burnout in terms of three core dimensions: emotional exhaustion (EE), depersonalization (DP), and reduced personal accomplishment (DA) (23). Related perspectives, including the emotional labor approach, further suggest that sustained role demands and resource dependence can intensify fatigue and reduce job satisfaction (JS).

**Figure 1:**
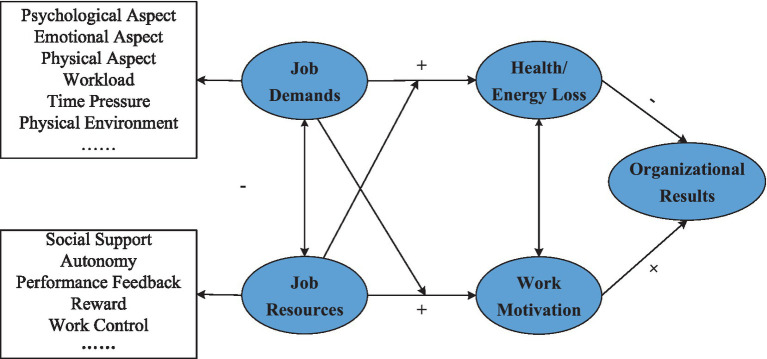
The job demands-resources model, based on Demerouti et al. (24) and Bakker and Demerouti (25).

The job demands-resources (JD-R) model (24–26) classifies work characteristics into job demands and job resources (JR) and has been widely used to explain employee health, motivation, and burnout outcomes (Figure 1) (27). Job demands refer to aspects of work that require sustained physical, cognitive, or emotional effort, such as workload, time pressure, role conflict, emotional labor, and performance expectations. JR, by contrast, refer to institutional and personal assets that help employees achieve work goals, reduce the costs of job demands, and support learning and development. These resources may include organizational support, managerial attention, incentives, feedback, autonomy, and opportunities for growth (24). In the context of rapid digital transformation, digital resources (DR) also affect work processes and occupational well-being (28). Prior research suggests that digital capabilities can strengthen human resource management and improve work support systems (29). In this study, however, DR are analytically distinguished from broader JR: JR mainly refer to institutionally provided support and incentives, whereas DR capture the technological capacities and digital support conditions that may reshape how job demands and JR operate.

**Figure 2:**
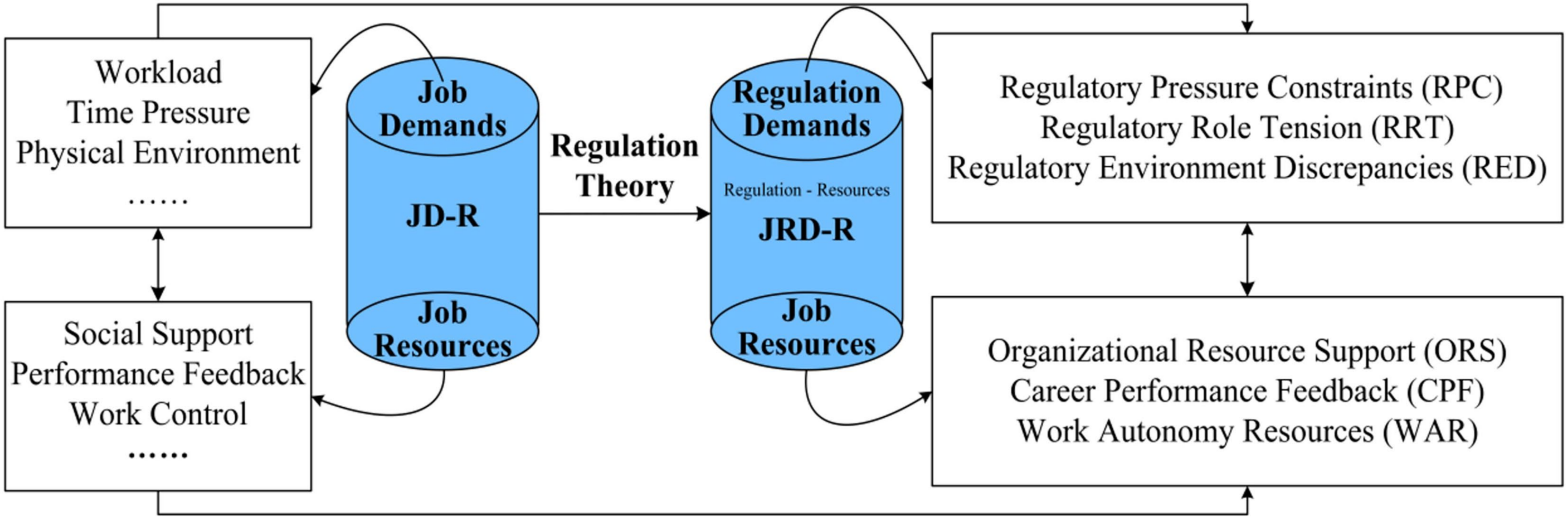
Theoretical analysis framework of “regulation-resources” based on the JD-R model.

One advantage of the JD-R perspective is its flexibility in adapting the concepts of demands and resources to different occupational settings (24). This flexibility makes the model especially suitable for examining grassroots traffic auxiliary police, whose work is embedded in a highly institutionalized and rule-bound environment. Regulatory theory (17, 18) provides a useful lens for extending the demand side of the JD-R model because it highlights how rules, procedures, supervisory control, and accountability mechanisms structure workplace behavior. Drawing on the Job Demand-Control and Demand-Control-Support traditions, this study develops a revised “regulation-resources” framework (Figure 2). In this framework, regulation corresponds to job demands and is conceptualized as job regulation demands (JRD), namely, work pressure generated by institutional rules, hierarchical supervision, procedural accountability, and performance assessment. More specifically, we distinguish among three dimensions: regulatory pressure constraints (RPC), regulatory role tension (RRT), and regulatory environment discrepancies (RED). These dimensions capture, respectively, the direct constraints imposed by rules and supervision, the conflicts created by multiple role expectations, and the strain produced by working across heterogeneous regulatory settings such as work-family boundaries. This concept of JRD is not identical to red tape (3), bureaucratic burden (19), or administrative burden (20, 21). Rather, it refers to institutionalized work requirements that are specific to auxiliary law enforcement, including mandatory orders, standardized procedures, and soft constraints imposed by the broader regulatory environment. By contrast, JR refer to the formal and informal resources available to employees, including organizational resource support (ORS), career performance feedback (CPF), and work autonomy resources (WAR). These concepts provide the basis for the hypotheses developed below.

### 2.2 Hypotheses

#### 2.2.1 Job regulation demands and job burnout

Regulatory theory emphasizes the role of rules, supervisory requirements, and institutional control in shaping behavior (30). Within pressure-based administrative systems, rules do not simply coordinate work; they also create psychological demands by tightening accountability, intensifying monitoring, and compressing time and performance expectations (17). Existing studies show that moderate levels of time pressure may sometimes promote goal orientation, task performance, and subjective well-being (31). However, when regulatory demands become excessive, they consume personal energy, restrict employees’ capacity to invest effort strategically, and increase the risk of exhaustion and disengagement. Likewise, role conflict, role ambiguity, and boundary-spanning role expectations have been linked to lower job performance and JS (32). Research based on person-environment fit also suggests that stress is likely to intensify when employees’ abilities and needs are poorly matched with work requirements and environmental conditions. High job demands coupled with insufficient resources can spill over into work-family interference and broader psychological strain (33).

Existing studies indicate that regulatory demands are shaped by multiple factors, especially regulatory pressure, role tension, and environmental discrepancies. First, RPC refer to the compliance burden produced by dense rules, target responsibility systems, inspections, performance evaluation, and continuous accountability transmission. Research on administrative burden and digital burnout in grassroots governance suggests that pressure-based systems can raise compliance, learning, and psychological costs, especially when digital tools are layered onto already demanding bureaucratic routines (20). In police settings, organizational stressors tend to predict stress reactions more strongly than operational hazards. Among police officers, the most highly rated and most frequently occurring stressors are not physical threats, but organizational demands, including excessive paperwork, bureaucratic procedural constraints, and rigid chain-of-command requirements (34). These findings suggest that rules and procedures designed to ensure accountability may gradually accumulate as chronic organizational stressors when adequate structural support is lacking. For auxiliary police officers who lack the full institutional protections afforded to formal police officers, such constraints are particularly likely to be perceived as burdens, thereby reinforcing the transmission pathway from regulatory demands to burnout. These pressures are further reinforced by strict legal and organizational control, contested authority boundaries, and persistent staffing and fiscal constraints (12–14).

Second, RRT captures the strain generated by incompatible role expectations and role ambiguity. Role conflict and role ambiguity are significant predictors of EE and DP among police officers, and these effects remain even after controlling for workload (35). Value discrepancy—that is, the conflict between police professional values and organizational role expectations—significantly predicts job burnout and job dissatisfaction (36). For traffic auxiliary police, the gap between their formal status and frontline duties itself constitutes a form of structural regulatory pressure. Grassroots traffic auxiliary police are expected to obey formal police command, maintain public order, provide service-oriented assistance, and, in practice, sometimes handle quasi-independent frontline tasks. This creates tension between formal auxiliary status and substantive task expectations, as well as between limited authority and substantial responsibility (13, 14).

Third, RED refer to mismatches between workers’ capacities and the heterogeneous environments in which regulation is enacted, including work-family boundaries, online-offline supervision, and the tension between formal institutional requirements and semi-formal employment conditions. JD-R research has identified work–family conflict as a salient job demand, and recent studies of digital governance suggest that digital control, hidden overtime, and information overload can further intensify psychological strain and EE (20, 37, 38). Furthermore, work–family conflict is often identified as a major risk factor for burnout among police populations. It predicts EE over time and subsequent DP more strongly than general job demands (39). This evidence reveals a vicious cycle between RED and burnout: the spillover effect of institutional pressures into family life generates chronic emotional resource depletion. For traffic auxiliary police, who often face irregular shift schedules, emergency task assignments, and blurred boundaries between online and offline supervision, such discrepancies constitute a persistent source of psychological stress.

These arguments are particularly relevant to grassroots traffic auxiliary police (40), whose work is marked by sudden task assignments, intensive duty schedules, strict supervision, and persistent fiscal and institutional constraints. In such settings, regulatory pressure may contribute to burnout by producing emotional depletion, indifference toward work, and a diminished sense of professional accomplishment (13). Previous research in transportation-related occupations has likewise shown that work pressure can increase dissatisfaction and work alienation (41). In addition, stigmatized or negatively stereotyped perceptions of law enforcement personnel may further amplify burnout risks by shaping how regulatory demands are experienced and interpreted (42). For traffic auxiliary police, burnout should therefore be understood not only as a response to generic workload, but also as a response to institutionally embedded pressure, contradictory role expectations, and mismatched regulatory environments.

The above discussion indicates that differences in job burnout among grassroots traffic auxiliary police are closely associated with regulatory demands. Therefore, Hypothesis 1 proposes that groups subject to stringent regulatory constraints tend to bear a higher risk of job burnout.

*H1*: Job regulation demands are positively associated with job burnout among grassroots traffic auxiliary police. Specifically, greater regulatory pressure constraints, stronger role tension, and larger regulatory environment discrepancies are expected to increase emotional exhaustion, depersonalization, and reduced personal accomplishment.

#### 2.2.2 Job resources and job burnout

JR are central to employee well-being, motivation, and burnout prevention within the JD-R framework (33). According to Conservation of Resources theory, individuals are more resilient to stress when they can acquire, maintain, and mobilize valued resources. Evidence from policing and other high-strain public-service occupations further suggests that burnout is shaped not only by excessive demands but also by access to supportive and enabling work resources, including organizational support, recognition, recovery opportunities, and role-related discretion (6, 11, 43–45). Evidence from police samples supports these relationships. Organizational support and autonomy are the strongest negative correlates of burnout across multiple police studies (46). Police job resources buffer the positive effects of work stress on emotional exhaustion and depersonalization. These studies provide population-specific evidence suggesting that job resources may help reduce burnout risk among police populations, supporting the applicability of the JD-R model to this occupational group. Furthermore, for grassroots traffic auxiliary police, who work under strict hierarchical control while occupying a relatively insecure and weakly resourced occupational position, the availability of such resources is likely to be especially important for protecting occupational well-being and reducing burnout.

Generally, JR are conceptualized through three complementary subdimensions: ORS, CPF, and WAR. ORS captures perceived support, recognition, and assistance from the organization, supervisors, and colleagues, and thus reflects a core form of social-organizational resource in the JD-R literature (25). The appropriateness of ORS as a job resource for police has been supported by empirical evidence. Most studies suggest that perceived organizational support is a key determinant of employee well-being and stress reduction (47), significantly reducing emotional exhaustion and depersonalization while increasing work engagement (48). Although these findings span multiple occupational groups, their relevance to policing is clear: for traffic auxiliary police, whose institutional status is often marginalized and legal standing ambiguous, organizational support conveys a signal of inclusion and valued recognition.

CPF reflects whether auxiliary police receive fair evaluation, reward signals, and performance-related recognition that affirm the value of their work; in this sense, it represents a motivational and evaluative resource that may buffer strain and sustain engagement (44). The role of CPF as a unique job resource for police is strongly supported by the Effort-Reward Imbalance (ERI) model. High effort combined with low reward significantly increases the risk of burnout and mental health problems across various occupational groups, including emergency service personnel (49). Within the policing field, perceived procedural fairness in performance evaluation systems is crucial for maintaining police morale and reducing cynicism (50). For traffic auxiliary police, whose career paths and compensation are often less clear than those of formal police officers, fair and developmental performance feedback is a key resource for sustaining work motivation and professional fulfillment.

WAR captures the degree of discretion that auxiliary police retain over task arrangement and work execution within a highly standardized command structure. Prior longitudinal evidence suggests that work autonomy resources may function as an antecedent of lower emotional exhaustion and higher work engagement (51). Among police populations, work autonomy resources remains a significant negative predictor of burnout even after controlling for other job resources and demands (46). For traffic auxiliary police working under a strict paramilitary command structure, even modest task-level discretion—such as autonomously managing daily patrol rhythms or exercising judgment in handling minor violations—may remain a psychologically meaningful resource by strengthening competence, control, and occupational identity (13, 45).

*H2*: Job resources are negatively associated with job burnout among grassroots traffic auxiliary police. Specifically, organizational resource support, career performance feedback, and work autonomy resources are expected to reduce emotional exhaustion, depersonalization, and reduced personal accomplishment.

#### 2.2.3 The mediating role of job satisfaction

JS refers to employees’ overall evaluative judgment of their work and work environment (52), including perceptions of task value, recognition, fairness, and professional fulfillment. It is closely linked to burnout, especially emotional exhaustion and a reduced sense of accomplishment. Classic perspectives such as Human Relations Theory, Herzberg’s Two-Factor Theory, and Self-Determination Theory all suggest that employees’ satisfaction depends in part on whether work environments provide support, recognition, competence, autonomy, and relatedness. These perspectives align with the JD-R framework, in which the balance between demands and resources shapes both psychological need satisfaction and occupational well-being (44). In general, high demands combined with inadequate support tend to depress JS, whereas organizational support, career-related recognition, and perceived opportunities for growth tend to enhance it (44, 53). Evidence from Chinese occupational settings likewise suggests that work stress and burnout are closely associated with lower JS and poorer work outcomes (54).

Prior research further suggests that the relationship between JS and burnout may be reciprocal over time. Some studies emphasize that burnout erodes JS, whereas others show that more positive work evaluations may buffer emotional exhaustion and disengagement (44, 52, 55). For example, research on employees’ perceptions of workplace ICTs shows that higher burnout is associated with lower JS, indicating that burnout may act as a downstream driver of dissatisfaction under conditions of technological and work-family strain (55). Related evidence also suggests that burnout can mediate the negative effect of work stress on JS, meaning that high work stress first intensifies burnout and then lowers satisfaction (54). At the same time, studies grounded in the JD-R and need-satisfaction traditions indicate that higher JS may protect against emotional exhaustion, depersonalization, and reduced accomplishment by strengthening occupational identification, fairness perceptions, and emotional self-regulation (44, 52).

Therefore, JS is treated not as the only possible consequence of burnout, but as a proximal evaluative mechanism through which institutional demands and resources are translated into burnout-related outcomes. This modeling choice follows the logic of the JD-R and need-satisfaction literature: high regulatory pressure and insufficient resources are likely to reduce satisfaction with one’s work, and lower satisfaction may in turn intensify EE and work withdrawal, whereas higher satisfaction may buffer burnout by reinforcing occupational identity, meaningfulness, and emotional regulation capacity. This mediating logic is especially relevant for grassroots traffic auxiliary police, whose semi-formal status, limited recognition, constrained career prospects, and resource insecurity may make everyday work evaluations particularly sensitive to regulatory pressure and support conditions (13, 14, 54). Accordingly, drawing on regulation theory and prior research on the relationships among the JD-R framework, JS, and burnout, we propose the following hypotheses:

*H3a*: Job regulation demands are negatively associated with job satisfaction, whereas job resources are positively associated with job satisfaction.

*H3b*: Higher job satisfaction is associated with lower job burnout among grassroots traffic auxiliary police.

*H3c*: Job satisfaction mediates the relationship between job regulation demands and job burnout and mediates the relationship between job resources and job burnout among grassroots traffic auxiliary police.

#### 2.2.4 The moderating role of digital resources

DR have become increasingly important in contemporary workplaces and are especially salient in the context of digital transformation in grassroots governance. From a resource dependence perspective, organizations rely on access to critical resources—including technical infrastructure, digital knowledge, leadership support, and data capabilities—to sustain operations and adapt to changing environments. Existing studies suggest that DR can have dual effects. On the one hand, they may enhance efficiency, communication, and coordination. On the other hand, when employees face technological requirements that exceed their skills or support conditions, digitalization may produce techno-stress, digital burnout, and broader psychological costs (56–58).

Recent research also shows that DR and digital support systems can reshape job demands, technostress, and access to work-related support (57–59). Recent Chinese public-administration research likewise suggests that digital governance may increase compliance, learning, and psychological costs under pressure-based systems, thereby contributing to digital burnout when technology becomes an additional layer of control rather than support (20). In the context of policing, DR may therefore shape how auxiliary police experience both regulation and institutional support. Adequate DR may make work rules more manageable, improve information processing, and strengthen the effectiveness of JR. By contrast, weak or poorly matched digital support may create additional burdens, reinforce monitoring pressure, and intensify the strain associated with digitalized work processes (59, 60).

Accordingly, recent research suggests that the role of DR is not merely direct but also contingent. In AI-enabled and digitally intensive workplaces, DR can relieve technostress and burnout by improving information access, task coordination, skill support, and perceived control; at the same time, they may create new burdens when digitalization increases constant connectivity, compliance work, surveillance, or cognitive overload (20, 29, 37, 55, 57–60). The key analytical issue is therefore whether DR alter the strength with which job demands translate into strain and JR translate into protection. For grassroots traffic auxiliary police, digital tools such as integrated traffic-management platforms, mobile terminals, body-worn and AI-assisted devices, and data-sharing systems may optimize regulatory enforcement, accelerate information verification, and improve the efficiency with which organizational support, feedback, and work coordination are accessed. Under stronger digital-resource conditions, regulatory demands may become easier to manage and institutional resources may be more efficiently converted into work support and reduced burnout. Under weaker, poorly matched, or more controlling digital conditions, however, the same regulatory and organizational arrangements may generate stronger fatigue responses by adding technostress, hidden compliance work, and digital monitoring pressure, particularly under the paradoxes of digital transformation (61). On this basis, the following hypotheses are proposed:

*H4a*: Digital resources moderate the relationship between job regulation demands and job burnout among grassroots traffic auxiliary police.

*H4b*: Digital resources moderate the relationship between job resources and job burnout among grassroots traffic auxiliary police.

**Figure 3:**
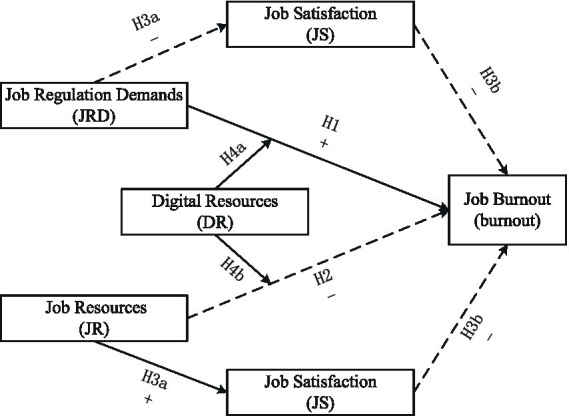
Conceptual model of the hypothesized relationships. Solid lines indicate positive relationships, whereas dashed lines indicate negative relationships.

Based on these hypotheses, Figure 3 presents the conceptual relationships among the study variables.

## 3 Materials and methods

### 3.1 Sampling and data collection

This study examines job burnout among grassroots traffic auxiliary police in G City, southern China. This group provides an appropriate setting for testing the proposed framework for two reasons. First, the work of traffic auxiliary police is shaped by both formal regulatory requirements and uneven access to organizational resources, making them well suited for a study of JRD and JR. Second, auxiliary police constitute a large non-established workforce in China, and their employment conditions reflect broader tensions in grassroots public-sector staffing, including high task intensity, limited career security, and constrained resource support.

A multistage stratified sampling design was used to improve coverage and heterogeneity. In the first stage, districts were sampled across the study area according to administrative divisions. In the second stage, subdistricts were selected within each sampled district. In the third stage, respondents were proportionally sampled within selected subdistricts by key demographic characteristics, including age and gender. To complement the survey data, purposive and convenience sampling were used to recruit auxiliary police officers and relevant practitioners for semi-structured interviews and expert consultations. Sample-size estimation was conducted in G*Power 3.1 using a small-to-moderate effect size (f^2^ = 0.10), *α* = 0.05, and power = 0.80. The resulting minimum sample size was 498, and the target sample size was increased to 530 to allow for invalid responses and fieldwork attrition.

The questionnaire was developed through a standard scale-development process, including construct clarification, item generation, expert review, pilot testing, and reliability and validity assessment. Item design drew on established measures of burnout (62), the JD-R framework (24, 63), JS (3, 64), and DR (57, 65, 66), and was further adapted to the occupational setting of grassroots traffic auxiliary police. Experts in human resource management, public security management, public health, and grassroots governance assessed item importance, relevance, and clarity. All items met the content-validity threshold (CVI > 0.78). A pilot survey of 35 traffic auxiliary police officers was then conducted, and the final instrument was refined based on pilot responses and interview feedback. Confirmatory factor analysis (CFA) showed that the measurement model had acceptable fit: χ^2^/df = 2.17, CFI = 0.934, TLI = 0.921, RMSEA = 0.072 (90% CI: 0.065–0.079), and SRMR = 0.068. Across the main constructs, Cronbach’s *α* ranged from 0.817 to 0.948, composite reliability (CR) ranged from 0.824 to 0.951, and average variance extracted (AVE) ranged from 0.512 to 0.827, indicating good reliability and convergent validity. A summary of these reliability and validity indices is provided in Appendix 2, and the heterotrait-monotrait ratio (HTMT) results for the core latent constructs are reported in Appendix 3. Formal data collection proceeded in two stages. In June 2025, the research team conducted fieldwork, including a symposium supported by the traffic management department, to refine the questionnaire and collect preliminary information. A second-round online survey was administered in November–December 2025 by three trained interviewers. The full questionnaire contained 40 items in total, including the core scale items reported in Appendix 1 and additional non-core background or fieldwork items that were not entered as main latent constructs; 534 responses were collected. After excluding invalid responses, including patterned responses, logically inconsistent responses, and questionnaires with missing or unusable information, 502 valid cases were retained, yielding an effective response rate of 94.0%.

The final sample was socio-demographically diverse (Table 1). Most respondents were male (64.8%), concentrated in the 30–45 age range, and mainly educated at the high-school/vocational or college/undergraduate level. More than half held lower-ranking positions. In this study, “rank” refers to the hierarchical grade within the auxiliary police personnel system (e.g., from junior auxiliary officer to auxiliary police supervisor), as defined by the local public security bureau. Respondents self-reported their grade on a five-level scale (1 = Low to 5 = High). Existing research suggests that higher-ranked officers typically have greater job security, more autonomy, and better access to institutional resources, which may reduce burnout risk. To standardize data collection, all interviewers received training on the study objectives, survey procedures, and ethical requirements before the anonymous administration of the questionnaire.

**Table 1:** Sample distribution (*N* = 502).

Variables	Assignment	Frequency (*n*)	Percentage (%)
Gender	1 = Female	177	35.2
	2 = Male	325	64.8
Age	1 = 18–30 years old	92	18.3
	2 = 30–40 years old	178	35.5
	3 = 40–50 years old	156	31.1
	4 = 50–60 years old	68	13.5
	5 = Over 60 years old	8	1.6
Education	1 = Primary school and below	10	2
	2 = Junior high school	38	7.6
	3 = Senior high school/Technical secondary school	162	32.3
	4 = College/Bachelor’s degree	238	47.4
	5 = Postgraduate degree	54	10.7
Rank	1 = Low	138	27.5
	2 = Relatively low	142	28.3
	3 = Moderate/Medium	122	24.3
	4 = Relatively high	70	13.9
	5 = High	30	6

Rank refers to the respondent’s self-reported hierarchical grade within the auxiliary police system (1 = Low to 5 = High).

### 3.2 Variables and measurements

All multi-item measures were translated using a translation/back-translation procedure (67) and then reviewed by researchers familiar with burnout research and the JD-R literature, together with experts in occupational mental health, human resource management, public security management, and grassroots governance. Minor wording adjustments were made to improve semantic equivalence and contextual fit for Chinese traffic auxiliary police. The study included measures of job burnout, JRD, JR, JS, DR, and demographic characteristics. Unless otherwise noted, the questionnaire used a five-point Likert scale ranging from 1 (strongly disagree) to 5 (strongly agree).

#### 3.2.1 Dependent variable: job burnout

Job burnout was operationalized as a three-dimensional construct comprising EE, DP, and DA (16, 23, 62). These three dimensions jointly capture the burnout profile of grassroots traffic auxiliary police. EE reflects sustained emotional depletion; DP captures detached or cynical responses to work; and DA reflects a reduced sense of efficacy and accomplishment.

Drawing on the Maslach Burnout Inventory (MBI), the Copenhagen Burnout Inventory (CBI), the Oldenburg Burnout Inventory (OLBI), and prior Chinese burnout research on grassroots public employees (62, 68), this study constructed a context-specific burnout scale suited to the occupational environment of traffic auxiliary police.

The burnout scale contained 12 items. Example items included “Work makes me feel physically and mentally exhausted” (EE; 5 items, *α* = 0.948), “I am starting to doubt the meaning of my work” (DP; 3 items, α = 0.904), and “I am able to use my professional expertise in my job” (DA; 4 items, α = 0.905; reverse-coded). Responses were recorded on a five-point scale ranging from 1 (never) to 5 (almost daily), with higher scores indicating higher burnout. The three dimensions all showed excellent internal consistency (*α* > 0.90). The overall KMO value was 0.949, and the cumulative explained variance reached 85.013%, indicating strong factorial adequacy. All items loaded above 0.50 on their intended factors. In addition, the measurement model showed acceptable fit (CFI and TLI ≥ 0.90; RMSEA and SRMR = 0.07–0.08) (69). The overall burnout scale also showed Cronbach’s α = 0.942, CR = 0.945, and AVE = 0.543 in the current *N* = 502 cohort.

#### 3.2.2 Primary independent variables: JRD and JR

The main predictors were JRD and JR. JRD comprised RPC, RRT, and RED. JR comprised ORS, CPF, and WAR. Together, the two constructs were measured with 17 items. All items were rated on a five-point Likert scale (1 = strongly disagree, 5 = strongly agree). Respondents were asked to assess the pressures and resources they had experienced in their daily work. Across these predictors, Cronbach’s *α* ranged from 0.817 to 0.936, the overall KMO value was 0.923, all factor loadings exceeded 0.50, average variance extracted values were above 0.50, and composite reliability values were above 0.80, indicating good reliability and convergent validity. These dimension-level reliability and validity results were calculated from the final *N* = 502 sample and are reported in Appendix 2, 3.

##### 3.2.2.1 Job regulation demands

JRD captures institutionalized job demands arising from formal rules, supervisory monitoring, accountability requirements, and heterogeneous enforcement contexts. For traffic auxiliary police, these demands are expressed through task overload, role ambiguity or conflict, contested authority boundaries, emergency enforcement duties, and tension between work and non-work domains. Drawing on research on work stress (70), role overload (71), and job demands, together with insights from the interviews, eight items were developed to measure the JRD dimensions. Example items included “Organizational regulations generate many emergency traffic-management tasks, inspection assignments, or accountability-related duties, creating substantial pressure for me” and “I feel unclear about the accountability boundaries between regular police officers, auxiliary police officers, and my own work role.” Importantly, JRD is not used as a synonym for general red tape or administrative burden. RPC captures pressure created by law-enforcement rules, emergency assignments, inspections, and accountability transmission; RRT captures uncertainty and conflict between auxiliary status, command obedience, responsibility boundaries, and frontline enforcement expectations; and RED captures discrepancies between institutional enforcement settings and non-work domains, including shift-based, online-offline, and emergency-duty arrangements. All JRD subscales showed Cronbach’s *α* values above 0.80, CR values above 0.80, and AVE values above 0.50 in the current *N* = 502 cohort, indicating satisfactory reliability and convergent validity.

##### 3.2.2.2 Job resources

JR refers to the institutional and interpersonal resources available to traffic auxiliary police at work. In this study, JR included ORS, CPF, and WAR. The items were developed with reference to JD-R research (26, 63) on organizational support, motivational resources (44), autonomy, and policing contexts (45). Example items captured whether respondents felt that their work was recognized by the organization. Importantly, this construct focused on institutional JR and therefore did not include DR, which were treated separately as a moderator. The overall JR scale showed Cronbach’s *α* = 0.918, CR = 0.921, and AVE = 0.564.

#### 3.2.3 Mediating variable: job satisfaction

JS was measured as an overall evaluative orientation toward one’s job and work environment (3, 53). Drawing on the Michigan Organizational Assessment Questionnaire (64), the scale included three items covering perceived work gains, satisfaction with the work environment, and overall JS. A representative item was “Overall, I am very satisfied with my current work.” All items were rated on a five-point Likert scale (1 = strongly disagree, 5 = strongly agree). Within the current *N* = 502 cohort, the JS scale showed Cronbach’s *α* = 0.831, CR = 0.836, and AVE = 0.630, indicating acceptable reliability and convergent validity.

#### 3.2.4 Moderating variable: digital resources

DR captured respondents’ perceived access to and effective use of digital tools in daily work. The construct was measured using two items adapted from prior research on DR (66), technostress, and digital transformation in work settings (57, 60). A representative item was “I use intelligent tools in my daily work (e.g., police AI glasses).” Responses were recorded on a five-point scale. Although digital tools can be viewed as a type of job resource, this study treated DR as a distinct construct to capture technology-related resource investment and capability use under digital transformation. This distinction also helped reduce conceptual overlap with the broader JR measure. Within the current *N* = 502 cohort, the DR scale showed Cronbach’s *α* = 0.865, CR = 0.870, and AVE = 0.763, indicating acceptable reliability and convergent validity.

#### 3.2.5 Control variables: demographic characteristics

To reduce potential confounding, the analyses controlled for gender, age, educational attainment, and job rank. These variables were included because previous research suggests that burnout perceptions and work-related evaluations vary across demographic and occupational groups (68, 72). Control variables were coded according to their measurement level and entered into all multivariate models. Except for the control variables, all latent constructs were computed as the mean of the corresponding items and were evaluated through CFA.

### 3.3 Model construction and interpretation

Based on the hypotheses and variable design, the study employed multiple linear regression, mediation analysis, and moderation analysis. The direct associations between the regulation-resources framework and the burnout dimensions were estimated using multiple linear regression. Mediation was assessed through a sequence of regression models together with bootstrap estimation of indirect effects (73). Moderation was tested using hierarchical regression with interaction terms. The core equations are presented below:


$$\begin{array}{l} \mathrm{EE}/\mathrm{DP}/\mathrm{DA}=\beta_{0}+\beta_{1}\mathrm{RPC}+\beta_{2}\mathrm{RRT}+\beta_{3}\mathrm{RED}+\beta_{4}\mathrm{ORS}\\ +\beta_{5}\mathrm{CPF}+\beta_{6}\mathrm{WAR}+\beta_{7}\text{Gender}+\beta_{8}\mathrm{Age}\\ +\beta_{9}\text{Education}+\beta_{10}\text{Rank}+\varepsilon \end{array}$$ (1)

$$\text{Burnout}=\beta_{0}+\mathrm{\beta z}(\mathrm{JRD}/\mathrm{JR})+\sum \beta_{k}{\text{Control}}_{k}+\varepsilon_{1}$$ (2-1)

$$\mathrm{JS}=\beta_{0}+\mathrm{\beta a}(\mathrm{JRD}/\mathrm{JR})+\sum \beta_{k}{\text{Control}}_{k}+\varepsilon_{2}$$ (2-2)

$$\begin{array}{l} \text{Burnout}=\beta_{0}+\beta z^{'}(\mathrm{JRD}/\mathrm{JR})\\ +\mathrm{\beta b}\mathrm{JS}+\sum \beta_{k}{\text{Control}}_{k}+\varepsilon_{3} \end{array}$$ (2-3)

$$\text{Burnout}=\beta_{0}+\sum \beta_{k}{\text{Control}}_{k}+\varepsilon$$ (2-4)

$$\begin{array}{l} \text{Burnout}=\beta_{0}+\beta_{1}(\text{JRDc}/\mathrm{JRc})+\beta_{2}\mathrm{DRc}\\ +\sum \beta_{k}{\text{Control}}_{k}+\varepsilon \end{array}$$ (2-5)

$$\begin{array}{l} \text{Burnout}=\beta_{0}+\beta_{1}(\text{JRDc}/\mathrm{JRc})+\beta_{2}\mathrm{DRc}\\ +\beta_{3}[(\text{JRDc}/\mathrm{JRc})\times \mathrm{DRc}]+\sum \beta_{k}{\text{Control}}_{k}+\varepsilon \end{array}$$ (2-6)

In Equation 1, EE, DP, and DA are regressed on the JRD and JR dimensions and the control variables. R^2^ and adjusted R^2^ were used to assess model fit, F-tests were used to assess overall model significance, and standardized coefficients (β) were used to interpret effect direction and magnitude. Variance inflation factors (VIFs) were examined to assess multicollinearity.

Equations 2-1–2-3 represent the total-effect model, the mediator model, and the direct−/indirect-effect model, respectively. A mediation effect was inferred when the bootstrap confidence interval for the indirect effect did not include zero. When both the indirect effect and the residual direct effect were significant, the mediation was interpreted as partial; when only the indirect effect remained significant, the pattern was interpreted as full mediation.

Equations 2-4–2-6 represent the baseline model, the main-effects model, and the interaction model for moderation testing. JRDc, JRc, and DRc are mean-centered variables, and JRDc × DRc and JRc × DRc are the interaction terms. A statistically significant interaction coefficient indicates that DR moderate the relationship between JRD or JR and burnout. Changes in R^2^ and simple-slope analyses were used to interpret the direction and strength of the moderation effects.

### 3.4 Statistical analysis

All analyses were conducted in Stata/MP 18.0. The analytical procedure included four steps. First, descriptive statistics were used to summarize the sample and variable distributions. Second, Pearson correlations were estimated to examine bivariate associations among the core variables. Third, multiple linear regression models were used to test the direct effects of JRD and JR on burnout dimensions, with diagnostic checks for multicollinearity, heteroskedasticity, and model specification. The HTMT test further showed that all HTMT values ranged from 0.12 to 0.78 (the HTMT values of the core indicators are between 0.41 to 0.75, as shown in Appendix 3), which is below the acceptable threshold of 0.85, indicating good discriminant validity and suggesting that serious collinearity was unlikely. Fourth, mediation and moderation analyses were conducted to examine the role of JS and DR. All reported models included the control variables. Statistical significance was set at *p* < 0.05 (two-tailed).

## 4 Empirical results

### 4.1 Assessment of common method bias

Because all core variables were collected through a self-report questionnaire, common method bias was assessed before the hypothesis tests (74). Procedurally, the survey was anonymous and administered under standardized instructions to reduce evaluation apprehension and social desirability bias. Methodologically, Harman’s single-factor test showed that no single factor accounted for more than 50% of the variance, and all extracted factors had eigenvalues > 1. In addition, all VIF values were below 5. Taken together, these results suggest that common method variance and multicollinearity were unlikely to threaten the interpretation of the main findings.

### 4.2 Descriptive statistics and correlations

Demographic variables (gender, age, educational attainment, and job rank) were treated as key controls. Reporting their correlations with the core variables helps assess potential confounding, supports the plausibility of the regression models, and provides a useful reference for control selection in burnout and JD-R research. Table 2 reports the means, standard deviations, and bivariate correlations of the main variables. The mean scores of the core variables ranged from 1.52 to 3.41, with standard deviations between 0.50 and 1.18, indicating adequate dispersion and no obvious outliers. The mean level of overall job burnout was 2.92 (SD = 1.02), suggesting a moderate level of burnout among the respondents. Among the three dimensions, EE showed the highest mean (M = 3.08, SD = 1.05), followed by DP (M = 2.94, SD = 1.02) and reduced DA (M = 2.75, SD = 1.02). As expected, JRD was positively correlated with job burnout (r = 0.52) and negatively correlated with JS (r = −0.50) and DR (r = −0.25). JR was negatively correlated with JRD (r = −0.37) and job burnout (r = −0.48), but positively correlated with JS (r = 0.56) and DR (r = 0.38). JS was negatively correlated with burnout (r = −0.53), whereas DR was positively correlated with JS (r = 0.36) and negatively correlated with burnout (r = −0.32). These patterns are consistent with prior JD-R research linking demands to strain and resources to satisfaction and lower burnout (63, 72). Burnout also varied across some background characteristics. Age showed a weak positive correlation with burnout (r = 0.24), and interview evidence suggested that officers aged 35–45 reported relatively higher burnout. Educational attainment and job rank were both negatively correlated with burnout (|r| = 0.39–0.52, *p* < 0.001), indicating that respondents with higher education and higher rank tended to report lower burnout.

**Table 2:** Means, standard deviations, and correlations among the main variables.

Variables	M ± SD	1	2	3	4	5	6	7	8	9
1. Burnout	2.92 ± 1.02	1								
2. JRD	3.25 ± 0.95	0.52***	1							
3. JR	2.78 ± 0.85	−0.48***	−0.37***	1						
4. JS	2.84 ± 0.91	−0.53***	−0.50***	0.56***	1					
5. DR	3.02 ± 0.89	−0.32***	−0.25***	0.38***	0.36***	1				
6. Gender	1.52 ± 0.50	0.18**	0.08	−0.07	−0.10	−0.07	1			
7. Age	2.68 ± 1.15	0.24***	0.20***	−0.16**	−0.18**	−0.10	0.05	1		
8. Education	3.41 ± 1.07	−0.39***	−0.28***	0.33***	0.37***	0.22***	−0.08	−0.11	1	
9. Rank	2.85 ± 1.03	−0.52***	−0.32***	0.46***	0.49***	0.25***	−0.09	−0.13*	0.47***	1

Burnout = job burnout; JRD = job regulation demands; JR = job resources; JS = job satisfaction; DR = digital resources. N = 502. Gender was coded as 1 = female and 2 = male. All descriptive statistics are based on the cleaned data before grand-mean centering. *p < 0.05, **p < 0.01, ***p < 0.001 (two-tailed).

### 4.3 Regression, mediation, and moderation analyses

Multiple linear regression was used to test the hypothesized associations between the JRD and JR framework and job burnout. Mediation and moderation analyses were then conducted to examine the roles of JS and DR.

#### 4.3.1 Direct effects of job regulation demands and job resources on burnout dimensions

Although CR and AVE were used to confirm the measurement validity of the JRD and JR dimensions, dimension-level regressions provide a more fine-grained view of how different subdimensions of the independent variables are associated with specific burnout dimensions, thereby helping identify the most salient drivers. Therefore, Table 3 reports the effects of the JRD and JR dimensions on the three burnout dimensions after controlling for demographic variables. Model M1 explained 61.2% of the variance in EE (adjusted R^2^ = 0.612; *F* = 84.094, *p* < 0.001), indicating strong explanatory power. RPC (*β* = 0.399, t = 5.392, *p* < 0.001), RED (*β* = 0.304, t = 3.897, *p* < 0.001), and RRT (*β* = 0.158, t = 2.225, *p* < 0.05) were positively associated with EE. ORS was negatively associated with EE (*β* = −0.231, t = −3.448, *p* < 0.01), whereas CPF and WAR showed negative but non-significant coefficients. Overall, EE increased as regulatory demands intensified and tended to decrease as organizational support improved.

**Table 3:** Regression results for the effects of JRD and JR on burnout dimensions (*N* = 502).

(A) Models M1 (EE) and M2 (DP)						
Variables	M1 β	M1 SE	M1 t	M2 *β*	M2 SE	M2 t
RPC	0.399	0.074	5.392***	0.417	0.091	4.582***
RRT	0.158	0.071	2.225*	0.242	0.109	2.22*
RED	0.304	0.078	3.897***	0.294	0.084	3.5**
ORS	−0.231	0.067	−3.448**	−0.195	0.081	−2.407*
CPF	−0.149	0.079	−1.886	−0.036	0.095	−0.379
WAR	−0.085	0.064	−1.328	−0.01	0.678	−0.015
gender	0.034	0.262	0.13	−0.028	0.663	−0.042
age	−0.028	0.259	−0.108	0.162	0.069	2.348
education	0.177	0.27	0.656	0.194	0.376	0.516
rank	−0.063	0.368	−0.171	−0.039	0.466	−0.084
R^2^	0.625	—	—	0.453	—	—
Adjusted R^2^	0.612	—	—	0.437	—	—
F	84.094***	—	—	90.136***	—	—

(B) Models M3 (DA) and M4 (Burnout)						
Variables	M3 β	M3 SE	M3 t	M4 β	M4 SE	M4 t
RPC	0.227	0.088	2.58*	0.372	0.082	4.537***
RRT	0.179	0.108	1.657	0.145	0.089	1.629
RED	0.292	0.083	3.518***	0.281	0.08	3.513***
ORS	−0.041	0.084	−0.488	−0.196	0.072	−2.722**
CPF	−0.198	0.093	−2.129*	−0.164	0.085	−1.929
WAR	0.01	0.076	0.132	−0.098	0.07	−1.4
gender	0.025	0.061	0.41	0.079	0.186	0.425
age	−0.033	0.058	−0.569	0.062	0.174	0.356
education	0.118	0.056	2.107*	0.105	0.168	0.625
rank	0.135	0.057	2.368*	−0.148	0.194	−0.763
R^2^	0.437	—	—	0.582	—	—
Adjusted R^2^	0.42	—	—	0.568	—	—
F	67.925***	—	—	79.364***	—	—

EE = emotional exhaustion; DP = depersonalization; DA = reduced personal accomplishment; RPC = regulatory pressure constraints; RRT = regulatory role tension; RED = regulatory environment discrepancies; ORS = organizational resource support; CPF = career performance feedback; WAR = work autonomy resources. EE, DP, and DA are the three dimensions of job burnout. RPC, RRT, and RED are the three dimensions of JRD, whereas ORS, CPF, and WAR are the three dimensions of JR. Standardized coefficients (β), standard errors (SE), and t statistics are reported. Significance levels are indicated by asterisks. All VIF values were below 5. *p < 0.05, **p < 0.01, ***p < 0.001.

Model M2 examined DP. The model explained 45.3% of the variance in this outcome (R^2^ = 0.453; *F* = 90.136, *p* < 0.001). RPC (*β* = 0.417, t = 4.582, *p* < 0.001), RED (*β* = 0.294, t = 3.500, *p* < 0.01), and RRT (*β* = 0.242, t = 2.220, *p* < 0.05) were positively associated with DP. ORS was negatively associated with DP (*β* = −0.195, t = −2.407, *p* < 0.05), whereas CPF and WAR were not statistically significant. Substantively, these results suggest that DP is more sensitive to chronic regulatory pressure, role tension, and work-family misalignment than to performance feedback or perceived autonomy.

Model M3 examined reduced DA. The model explained 43.7% of the variance in reduced DA (R^2^ = 0.437; adjusted R^2^ = 0.420; *F* = 67.925, *p* < 0.001). RED (*β* = 0.292, t = 3.518, *p* < 0.001) and RPC (*β* = 0.227, t = 2.580, *p* < 0.05) were positively associated with reduced DA, whereas CPF was negatively associated with reduced DA (*β* = −0.198, t = −2.129, *p* < 0.05). RRT, ORS, and WAR were not statistically significant. Taken together, the direct-effect models show that regulatory demands were associated with higher burnout, whereas organizational and performance-related resources were associated with lower burnout in selected dimensions.

When overall job burnout was modeled as the dependent variable, RPC and RED were positively associated with burnout, whereas ORS was negatively associated with burnout. The coefficients of RRT, CPF, and WAR were in the expected directions but did not reach conventional significance. Together with the construct-level mediation models reported below, these results provide support for H1 and H2 while also showing that the determinants of burnout vary across subdimensions. The control variables showed some differences across the correlation and regression analyses. For example, job rank was negatively associated with overall burnout in the bivariate correlation analysis, but the coefficient became smaller and non-significant in the multivariate model, suggesting that part of the bivariate association was shared with the core explanatory variables. Job rank also showed a small positive association with reduced DA, which may reflect the added responsibility and performance pressure associated with higher positions. These differences do not weaken the main conclusions; rather, they suggest that the determinants of burnout vary somewhat across its subdimensions.

#### 4.3.2 Mediating role of job satisfaction

Mediation was tested using stepwise regression and bootstrap estimation (Tables 4–6). Before the mediator was included, JRD positively predicted burnout (*β* = 0.353, *p* < 0.001) and negatively predicted JS (*β* = −0.302, *p* < 0.001), whereas JR negatively predicted burnout (*β* = −0.324, *p* < 0.001) and positively predicted JS (*β* = 0.332, *p* < 0.001). After JS was added to the model, the direct effects of JRD (*β* = 0.246, *p* < 0.01) and JR (*β* = −0.206, *p* < 0.01) remained significant but became smaller in absolute magnitude. JS itself was negatively associated with burnout (*β* = −0.355, *p* < 0.001). These patterns indicate partial mediation and support H3a-H3c.

**Table 4:** Mediation results for the effect of JRD on job burnout through JS (*N* = 502).

Variables	Burnout	JS	Burnout
Gender	0.052 (0.031)	0.048 (0.033)	0.061 (0.029)*
Age	0.041 (0.035)	0.039 (0.034)	0.042 (0.034)
Education	0.038 (0.042)	0.087 (0.045)	0.041 (0.040)
Rank	−0.112 (0.053)*	−0.108 (0.055)	−0.121 (0.051)*
JRD	0.353 (0.054)***	−0.302 (0.045)***	0.246 (0.059)***
JS	—	—	−0.355 (0.042)***
R^2^	0.582	0.386	0.641
Adjusted R^2^	0.576	0.378	0.635
F	79.36***	48.25***	92.18***

Standardized coefficients (β) and standard errors (SE) are reported. *p < 0.05, **p < 0.01, ***p < 0.001.

**Table 5:** Mediation results for the effect of JR on job burnout through JS (*N* = 502).

Variables	Burnout	JS	Burnout
Gender	0.059 (0.032)	0.051 (0.033)	0.064 (0.029)*
Age	0.044 (0.034)	0.039 (0.033)	0.028 (0.033)
Education	0.078 (0.043)	0.087 (0.045)	0.083 (0.042)*
Rank	−0.142 (0.052)**	−0.138 (0.053)**	−0.151 (0.050)**
JR	−0.324 (0.058)***	0.332 (0.046)***	−0.206 (0.060)**
JS	—	—	−0.355 (0.042)***
R^2^	0.582	0.372	0.638
Adjusted R^2^	0.576	0.364	0.632
F	79.36***	45.12***	89.45***

*p < 0.05, **p < 0.01, ***p < 0.001.

**Table 6:** Total, direct, and indirect effects (*N* = 502).

Path	Effect	Effect size	SE	95% CI lower limit	95% CI upper limit	Relative effect value
JRD → JS → burnout	Direct effect	0.246***	0.059	0.130	0.362	69.69%
	Indirect effect	0.107**	0.032	0.044	0.170	30.31%
	Total effect	0.353***	0.054	0.247	0.459	—
JR → JS → burnout	Direct effect	−0.206**	0.060	−0.324	−0.088	63.58%
	Indirect effect	−0.118***	0.033	−0.183	−0.053	36.42%
	Total effect	−0.324***	0.058	−0.438	−0.210	—

Bootstrap results are based on 5,000 resamples. *p < 0.05, **p < 0.01, ***p < 0.001.

The bootstrap results (Table 6) further confirmed the mediation findings. For JRD, the total effect on burnout was 0.353 (SE = 0.054, 95% CI [0.247, 0.459]), the direct effect was 0.246 (SE = 0.059, 95% CI [0.130, 0.362]), and the indirect effect via JS was 0.107 (SE = 0.032, 95% CI [0.044, 0.170]). Because none of the confidence intervals included zero, JS significantly mediated the relationship between JRD and burnout, accounting for 30.31% of the total effect. For JR, both the direct effect (95% CI [−0.324, −0.088]) and the indirect effect through JS (95% CI [−0.183, −0.053]) were significant, and the mediated proportion was 36.42%. These results further support H3c.

#### 4.3.3 Moderating role of digital resources

Hierarchical regression with interaction terms was used to test whether DR moderated the relationships between the regulation-resources framework and burnout (Tables 7, 8). After mean-centering the relevant variables, JRD remained positively associated with burnout (*β* = 0.238, *p* < 0.01), and the interaction term JRDc × DRc was significant and negative (*β* = −0.065, *p* < 0.01). This indicates that higher DR buffered the positive association between regulatory demands and burnout. JR remained negatively associated with burnout (*β* = −0.197, *p* < 0.01), and the interaction term JRc × DRc was also significant (*β* = −0.058, *p* < 0.05), suggesting that DR strengthened the protective effect of JR.

**Table 7:** Moderation test results for digital resources (*N* = 502).

Variables	Burnout			
	β	SE	t	*p*
Constant	0.098*	0.045	2.18	0.030
Gender	0.062	0.063	0.98	0.328
Age	0.049	0.067	0.73	0.466
Education	0.087	0.080	1.09	0.276
Rank	−0.129*	0.057	−2.26	0.024
JRDc	0.238**	0.076	3.13	0.002
JRc	−0.197**	0.075	−2.63	0.009
DRc	−0.186**	0.067	−2.78	0.006
JRDc×DRc	−0.065**	0.023	−2.83	0.005
JRc × DRc	−0.058*	0.026	−2.23	0.026
R^2^	0.607	—	—	—
Adjusted R^2^	0.591	—	—	—
F	82.42***	—	—	< 0.001

c indicates mean-centering. *p < 0.05, **p < 0.01, ***p < 0.001.

**Table 8:** Simple-slope results for the moderating effect of DR (*N* = 502).

Path	DR level	JRD → Burnout/JR → Burnout					
		Effect size	SE	t	*p*	95% CI lower limit	95% CI upper limit
JRD → burnout	High (M + 1 SD)	0.173**	0.061	2.86	0.004	0.053	0.293
	Low (M - 1 SD)	0.303***	0.062	4.92	<0.001	0.181	0.425
JR → burnout	High (M + 1 SD)	−0.255***	0.061	−4.17	<0.001	−0.375	−0.135
	Low (M - 1 SD)	−0.139*	0.065	−2.15	0.032	−0.266	−0.012

The reported effect values are simple slopes. *p < 0.05, **p < 0.01, ***p < 0.001.

**Figure 4:**
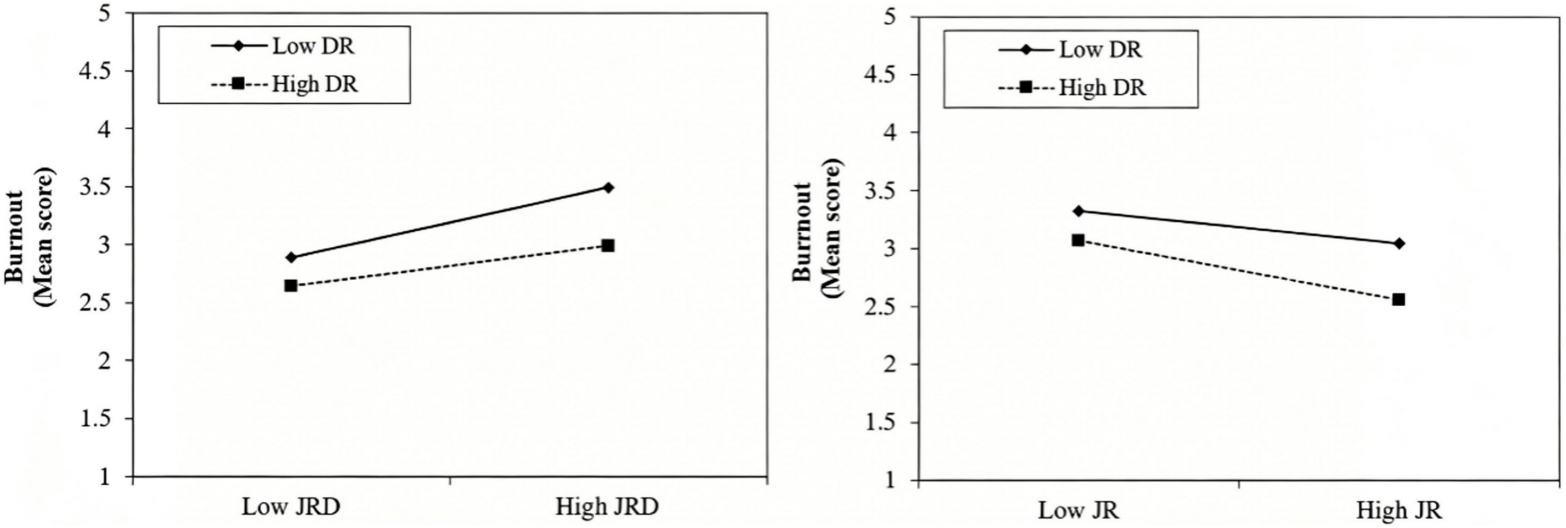
Moderating effects of digital resources.

Table 8 and Figure 4 present the simple-slope results. The moderation effects were significant at both low (M − 1 SD) and high (M + 1 SD) levels of DR (*p* < 0.05). Under low DR conditions, the positive association between JRD and burnout was stronger (effect = 0.303, 95% CI [0.181, 0.425]). By contrast, under high DR conditions, the adverse effect of JRD was weaker, and the burnout-reducing effect of JR was stronger. Importantly, after the moderating role of DR is considered, the effect of JRD on burnout remains positive, but its magnitude is substantially weakened. This pattern may reflect the way in which DR improve work efficiency, strengthen perceived support, and indirectly reduce burnout. Likewise, the more negative association between JR and burnout under high digital-resource conditions suggests a positive enabling effect of DR. In other words, the result should not be interpreted as indicating that both JRD and JR become negatively associated with burnout once DR are introduced. Rather, DR attenuate the harmful effect of JRD while strengthening the protective effect of JR. These findings support H4a and H4b.

## 5 Discussion

### 5.1 Main findings

The findings point to three main conclusions. First, the proposed JRD and JR framework is significantly associated with job burnout among grassroots traffic auxiliary police in China. Among the three burnout dimensions, EE shows the strongest association, followed by DP and reduced DA. This pattern suggests a progressive process in which sustained resource depletion first manifests as exhaustion, then erodes the meaning attached to work, and eventually weakens perceived professional efficacy. Burnout among traffic auxiliary police should therefore be understood not merely as an individual reaction to heavy work, but as a layered psychological process shaped by prolonged institutional pressure and uneven access to resources. This imbalance between regulatory demands and resource support also helps explain why burnout among auxiliary police is more than an individual adjustment problem; it is an occupational health issue rooted in organizational and institutional arrangements.

More specifically, the findings identify three salient regulatory stressors. First, RPC emerge as the strongest driver of burnout. Frequent inspections, strict accountability requirements, emergency response duties, and physically demanding outdoor work create chronic strain, especially for a workforce positioned outside the formal staffing system. Second, RRT is closely related to EE. Traffic auxiliary police are expected to remain disciplined and compliant while also responding flexibly to service-oriented and collaborative governance demands. These competing expectations consume cognitive and emotional resources and extend classic role-stress arguments to the context of semi-formal law enforcement. Third, RED—especially tensions between work and family, online and offline supervision, and formal and informal performance expectations—also contribute to burnout. The findings suggest that burnout is intensified not only by workload itself, but also by instability and inconsistency in the institutional settings in which work is performed.

Second, this study shows that JS serves as an important mediating mechanism linking the JRD and JR framework to job burnout. Rather than treating JS merely as an outcome, the findings indicate that it transmits the effects of both regulatory demands and JR. Higher regulatory pressure, stronger role tension, and greater RED are associated with lower JS, whereas stronger organizational support, more favorable career-related feedback, and greater work autonomy resources are associated with higher satisfaction. In turn, higher JS is related to lower burnout across EE, DP, and reduced DA. These results suggest that satisfaction-related experiences—such as fairness, recognition, belonging, and a sense of meaningful contribution—help auxiliary police sustain emotional self-regulation under demanding conditions.

Third, DR moderate the relationship between the JRD and JR framework and job burnout. The results indicate that stronger digital support and more effective use of digital tools can weaken the adverse effects of regulatory demands and strengthen the protective role of JR. In practical terms, DR appear to improve task coordination, information access, and perceived work efficiency, thereby helping auxiliary police cope with pressure, role ambiguity, and resource scarcity. At the same time, this finding should be interpreted cautiously. Digitalization can also generate new forms of monitoring pressure, technostress, and algorithmic control. The current results suggest that, in this sample, the benefits of digital support outweigh these costs, but that balance may change as digital management becomes more intensive. Future research should therefore continue to examine when DR function as supportive infrastructure and when they become an additional source of strain.

Our findings share both similarities and differences with previous research. Consistent with existing police burnout studies, organizational support and performance feedback were significant protective factors against burnout, and the overall pattern of job demands and resources jointly influencing burnout supports the cross-contextual applicability of the JD-R model in law enforcement populations (6–11). However, several differences emerged: First, work autonomy resources was not significantly associated with depersonalization or reduced personal accomplishment, contrasting with the robust protective effect reported in formal police studies (45), likely reflecting the structural vulnerability associated with auxiliary police officers’ semi-formal occupational status (75). Second, the pattern in which emotional exhaustion was more prominent than depersonalization and reduced personal accomplishment aligns with findings from other Chinese public sector samples (68), suggesting that resource depletion is the primary pathway through which work stress manifests. Third, the buffering effect of digital resources is consistent with recent evidence (61, 65) and is further extended in the highly regulated context of traffic policing. Fourth, the partial mediating effect of job satisfaction falls within the range reported across occupations (44), supporting satisfaction as a proximal evaluative pathway (52). In summary, this study’s findings have both common features and distinctive value.

### 5.2 Theoretical and practical implications

#### 5.2.1 Theoretical implications

First, by incorporating regulatory theory into the JD-R model, this study highlights the institutional foundations of burnout in grassroots collaborative law enforcement. Existing JD-R research has paid close attention to job demands and JR, but it has not always specified how formal rules, accountability mechanisms, and regulatory expectations shape those demands and resources in highly institutionalized settings. Our findings show that regulatory requirements are not merely background conditions; they are substantive features of the work environment that shape psychological strain, perceived fairness, and access to support. This perspective helps extend the JD-R framework from a general model of workplace stress to one that is more sensitive to institutional regulation, especially in public-sector occupations. Within public-health governance, the regulation-resources framework also offers a useful lens for explaining health inequality and psychological strain among frontline workers.

Second, the study offers a more integrated explanation of burnout by jointly examining regulation, resources, JS, and digitalization. Much of the existing literature has focused on broad work demands and resource availability, or has treated JS mainly as an outcome of burnout. By contrast, this study shows that JS operates as a meaningful psychological pathway, while DR condition the strength of the relationships between demands, resources, and burnout. In this respect, the study contributes to current debates on mental health in digitally mediated workplaces by showing that digitalization can simultaneously relieve and reshape work strain. It also provides a useful theoretical basis for strengthening digital mental-health support and reducing digital burnout in the course of everyday work.

Finally, this study extends burnout research to a group that has received comparatively limited attention in the literature: grassroots traffic auxiliary police. Most previous studies have focused on teachers, healthcare workers, and formally established police officers. By examining a large group of semi-formal public-sector workers, this study broadens the empirical scope of burnout research and adds to ongoing discussions about staffing dualism, occupational inequality, and the governance of non-established personnel. It also opens a new analytical perspective on labor-health equity, psychologically healthy law enforcement, and ethical practice among auxiliary police and related groups (76).

Taken together, these contributions suggest that burnout among auxiliary police should be analyzed not only at the individual level but also within a broader organizational and institutional context. The findings therefore provide an empirical basis for future research on burnout prevention among auxiliary police, including interventions related to self-regulation, team climate, organizational support, and the design of regulatory policy.

#### 5.2.2 Practical implications

The findings also carry practical implications for the professional development of auxiliary police, traffic governance, and the cultivation of psychological resilience among grassroots personnel. More broadly, they suggest that efforts to reduce burnout should not rely on individual coping alone. Instead, policy design, organizational support, satisfaction-oriented management, and appropriate digital empowerment need to be addressed in an integrated manner if institutions are to reduce mental health inequality and improve the sustainability of grassroots public employment.

Based on these findings, four practical recommendations can be proposed. First, regulatory arrangements should be redesigned to reduce unnecessary pressure while preserving organizational coordination. Clearer task allocation, early-warning mechanisms for high-risk duties, more reasonable shift schedules, and routine mental health assessment may help prevent frontline staff from becoming trapped in a cycle of chronic regulatory strain. Second, investment in JR should be strengthened. Organizational recognition, supportive supervision, fair performance feedback, and visible career incentives can improve occupational identification and buffer the negative effects of high demands. Third, management should place greater emphasis on JS as an operational target rather than treating it as a secondary consequence of burnout. Improvements in organizational communication, perceived fairness, and recognition may help sustain motivation and reduce psychological withdrawal. Fourth, digitalization should be implemented as a form of supportive empowerment rather than as an additional layer of surveillance. Better platform usability, digital training, and technologies that reduce repetitive work may help ensure that DR operate as protective rather than stress-inducing conditions.

Against the backdrop of global digital transformation, the present study also has practical and policy relevance. Policymakers can strengthen digital mental health interventions (77) and improve frontline work support through better digital systems, training, and leadership support (57, 59). Public institutions should invest in technical infrastructure, foster a supportive digital culture, and enhance auxiliary police officers’ digital literacy, ethical awareness, and digital resilience. Building healthier digital work environments (57) may reduce technostress while enabling digital tools to support routine patrol, investigation, traffic management, and public-order tasks, thereby improving task efficiency and occupational well-being. Digital monitoring and evaluation mechanisms should be designed to support, rather than overburden, frontline staff. Police-related agencies can also strengthen vocational training and organizational socialization to improve self-determination, professional identity, and long-term resilience (78).

This interpretation is broadly consistent with evidence from other Chinese public-sector occupations. Studies of government auditors and related personnel show that sustained work pressure is associated with stronger burnout, whereas relational and psychological resources can partially buffer, but not fully remove, strain (79, 80). Longitudinal JD-R evidence further suggests that work demands, work resources, and burnout may reinforce one another over time, underscoring the value of early organizational intervention before a self-reinforcing strain cycle is established (38).

### 5.3 Limits and future directions

This study has several limitations. First, the sample focuses on grassroots traffic auxiliary police in a single Chinese city. Although this group is important for understanding the occupational psychology of non-established personnel in grassroots governance, the generalizability of the findings to other categories of auxiliary police and other fiscally supported public-sector workers remains to be tested. Future studies could extend the analysis to other governance settings and adopt multisite or multisource designs. Second, the measurement of JRD can be further refined. Although the present study captures key aspects of institutional pressure, it may not fully represent the multidimensional character of regulatory work requirements across different formal and informal rule environments. Future research could therefore develop more differentiated measures of regulatory demands and incorporate subjective perceptions of compliance burden. Third, the study uses a cross-sectional design and primarily tests linear relationships. Although the analysis provides strong evidence of association, it cannot fully establish temporal ordering or nonlinear causal dynamics. In particular, the mediating role of JS should be interpreted as theory-driven rather than as definitive evidence of temporal causality. Longitudinal, panel, and quasi-experimental designs would help address these limitations and clarify whether changes in demands, resources, and burnout generate cumulative or feedback effects over time (38).

## 6 Conclusion

This study integrates the JD-R model with a regulatory perspective to develop a regulation-resources analytical framework and tests it using survey data from 502 traffic auxiliary police officers in G City, southern China. The findings show that JRD are positively associated with burnout, whereas JR are negatively associated with burnout. JS partially mediates these relationships, and DR moderate them. The results also suggest that burnout varies across demographic groups. Overall, the study underscores the importance of combining regulatory reform, resource support, satisfaction-oriented management, and carefully designed digital empowerment to protect the occupational health of grassroots auxiliary police.

## Data Availability

The datasets presented in this article are not publicly available because they contain occupational survey information from a sensitive occupational group and were collected under conditions of anonymity, confidentiality, and applicable institutional confidentiality requirements. De-identified and aggregated data may be made available by the corresponding author upon reasonable request, subject to approval by the relevant institution and in accordance with the informed consent provided by participants. Requests to access the datasets should be directed to Chengcheng Liu, lccEmma@outlook.com.

## References

[1] Gao D Zhang X Zhang X Ma J. Environmental regulation: an enhancing or burden for social welfare and public health? J Clean Prod. (2024) 441:140985. doi: 10.1016/j.jclepro.2024.140985

[2] Viscusi WK Vernon JM Harrington JE. Economics of Regulation and Antitrust. Cambridge, MA: The MIT Press (1995).

[3] Li Q George B. Untangling the relationship between red tape and job satisfaction: the role of self-efficacy and high-individualistic culture. Public Adm Rev. (2025) 85:1723–37. doi: 10.1111/puar.13940

[4] Kennedy N Win TL Bandyopadhyay A Kennedy J Rowe B McNerney C . Insights from linking police domestic abuse data and health data in South Wales, UK: a linked routine data analysis using decision tree classification. Lancet Public Health. (2023) 8:e629–38. doi: 10.1016/S2468-2667(23)00126-3, 37516479

[5] Parfitt R Pantaleão B Kopittke A. Police autonomy, data-driven strategies, and violence: evidence from Brazil's policing reform. J Dev Econ. (2026) 178:103603. doi: 10.1016/j.jdeveco.2025.103603

[6] Alves L Abreo L Petkari E Pinto da Costa M. Psychosocial risk and protective factors associated with burnout in police officers: a systematic review. J Affect Disord. (2023) 332:283–98. doi: 10.1016/j.jad.2023.03.081, 36972850

[7] Zou H. The theoretical paradox of auxiliary police in China and its resolution: starting from the first local government regulation on auxiliary police. Acad Forum. (2012) 35:61–6. doi: 10.16524/j.45-1002.2012.11.027

[8] Zhang H. Subject positioning and norms of auxiliary police. Sci Law. (2011) 9:124–31.

[9] Xu Z. The construction of auxiliary police organizations in the process of marketization in China. J People Public Secur Univ China. (2011) 27:143–7.

[10] Heath C. The special constable at the college of surgeons. Lancet. (1870) 95:638. doi: 10.1016/S0140-6736(02)66302-0

[11] Liang Z Yao M Li H Chen J Yang M Tang T . How does the double-track human resource management model contribute to job burnout and mental health among Chinese government departments? A Chinese police study. Front Public Health. (2024) 12:1423103. doi: 10.3389/fpubh.2024.1423103, 39301515 PMC11410598

[12] Yang Z Chen X. Soft constraints on personnel quota management or institutional flexibility: an empirical study based on the expansion of auxiliary police in four cities. Chinese journal Political Science Studies (政治学研究), (2019) 2:88–99.

[13] Zhao J. The indigenous and innovative dimensions of the rule of law in social governance: centering on the "independent policing" of traffic auxiliary police. Sci Law. (2019) 37:64–73. doi: 10.16290/j.cnki.1674-5205.2019.03.006

[14] Ma Y Huang J. Where to go: identity, emotion and ethical practice of career-track auxiliary police. J Hubei Univ National. (2024) 42:20–8. doi: 10.13501/j.cnki.42-1328/c.2024.03.001

[15] National Bureau of Statistics of China. China Statistical Yearbook 2025. Beijing: China Statistics Press (2025).

[16] Maslach C Jackson SE. The measurement of experienced burnout. J Organ Behav. (1981) 2:99–113. doi: 10.1002/job.4030020205

[17] Yu L Xiao X. Review on the development of regulation theory. Res Financial Econ Issues. (2001) 1:17–24. doi: 10.19654/j.cnki.cjwtyj.2001.01.003

[18] Meier K. The Political Economies of Regulation: The Case of Insurance. Albany, NY: State University of New York Press (1998).

[19] Gordon DM. Fat and Mean: The Corporate Squeeze of Working Americans and the Myth of Managerial “Downsizing”. New York, NY: The Free Press (1996).

[20] Shu Q Yang X Liu L. How does smart social governance generate digital burnout: an analysis based on administrative burden theory. Chinese Public Admin. (2025) 41:98–109. doi: 10.19735/j.issn.1006-0863.2025.01.11

[21] Burden BC Canon DT Mayer KR Moynihan DP. The effect of administrative burden on bureaucratic perception of policies: evidence from election administration. Public Adm Rev. (2012) 72:741–51. doi: 10.1111/j.1540-6210.2012.02600.x

[22] World Health Organization. "Burnout (Code QD85)". In: International Classification of Diseases 11th Revision. Geneva: World Health Organization (2019)

[23] Maslach C Schaufeli WB Leiter MP. Job burnout. Annu Rev Psychol. (2001) 52:397–422. doi: 10.1146/annurev.psych.52.1.397 11148311

[24] Demerouti E Bakker AB Nachreiner F Schaufeli WB. The job demands-resources model of burnout. J Appl Psychol. (2001) 86:499–512. doi: 10.1037/0021-9010.86.3.499, 11419809

[25] Bakker AB Demerouti E. The job demands-resources model: state of the art. J Manag Psychol. (2007) 22:309–28. doi: 10.1108/02683940710733115

[26] Demerouti E Bakker AB. Job demands-resources theory in times of crises: new propositions. Organ Psychol Rev. (2023) 13:209–36. doi: 10.1177/20413866221135022

[27] Bakker AB Demerouti E. Job demands-resources theory: frequently asked questions. J Occup Health Psychol. (2024) 29:188–200. doi: 10.1037/ocp0000376, 38913705

[28] Bennich A. The digital imperative: institutional pressures to digitalise. Technol Soc. (2024) 76:102436. doi: 10.1016/j.techsoc.2023.102436

[29] Deng C Li H Wang Y Zhu R. The double-edged sword in the digitalization of human resource management: person-environment fit perspective. J Bus Res. (2024) 180:114738. doi: 10.1016/j.jbusres.2024.114738

[30] Zhang L. Summary of research on government regulation theory in China. Chinese Public Admin. (2006) 12:87–90.

[31] Lu X Kluemper D Tu Y Zhou H. Goal orientation, time pressure, and daily job crafting profiles: an integration of job demands-resources model and approach-avoidance perspective. J Vocat Behav. (2025) 160:104128. doi: 10.1016/j.jvb.2025.104128

[32] Tubre TC Collins JM. Jackson and Schuler (1985) revisited: a meta-analysis of the relationships between role ambiguity, role conflict, and job performance. J Manag. (2000) 26:155–69. doi: 10.1177/014920630002600104

[33] Janssen PPM Peeters MCW De Jonge J Houkes I Tummers GER. Specific relationships between job demands, job resources and psychological outcomes and the mediating role of negative work-home interference. J Vocat Behav. (2004) 65:411–29. doi: 10.1016/j.jvb.2003.09.004

[34] Violanti JM Fekedulegn D Hartley TA Charles LE Andrew ME Ma CC . Highly rated and most frequent stressors among police officers: gender differences. Am J Crim Justice. (2016) 41:645–62. doi: 10.1007/s12103-016-9342-x, 28260848 PMC5330309

[35] Burke RJ. Work-family stress, conflict, coping, and burnout in police officers. Stress Med. (1993) 9:171–80. doi: 10.1002/smi.2460090308

[36] Schaible LM Gecas V. The impact of emotional labor and value dissonance on burnout among police officers. Police Q. (2010) 13:316–41. doi: 10.1177/1098611110373997

[37] Ninaus K Diehl S Terlutter R. Employee perceptions of information and communication technologies in work life, perceived burnout, job satisfaction and the role of work-family balance. J Bus Res. (2021) 136:652–66. doi: 10.1016/j.jbusres.2021.08.007

[38] Huang J Wu GQ Wang YS You XQ. Why I am becoming burned out? The role of changes in job demands and resources. Psychol Sci. (2015) 38:708–14. doi: 10.16719/j.cnki.1671-6981.2015.03.031

[39] Hall GB Dollard MF Tuckey MR Winefield AH Thompson BM. Job demands, work-family conflict, and emotional exhaustion in police officers: a longitudinal test of competing theories. J Occup Organ Psychol. (2010) 83:237–50. doi: 10.1348/096317908X401723

[40] Greenberg MA. Auxiliary Police: The Citizen’s Approach to Public Safety. Westport, CT: Greenwood Press (1984).

[41] Jou RC Kuo CW Tang ML. A study of job stress and turnover tendency among air traffic controllers: the mediating effects of job satisfaction. Transp Res Part E Logist Transp Rev. (2013) 57:95–104. doi: 10.1016/j.tre.2013.01.009

[42] Hassan S Wright BE Tinkler JE. Anticipated stigma and burnout: the impact of concerns about being perceived as racist among law enforcement officers. Public Adm Rev. (2026) 86:1106–22. doi: 10.1111/puar.70085

[43] Demerouti E. Job demands-resources and conservation of resources theories: how do they help to explain employee well-being and future job design. J Bus Res. (2025) 192:115296. doi: 10.1016/j.jbusres.2024.115296

[44] Morin AJS Gillet N Blais AR Comeau C Houle SA. A multilevel perspective on the role of job demands, job resources, and need satisfaction for employees' outcomes. J Vocat Behav. (2023) 141:103846. doi: 10.1016/j.jvb.2023.103846

[45] Hu Q Schaufeli WB Taris TW. How are changes in exposure to job demands and job resources related to burnout and engagement? A longitudinal study among Chinese nurses and police officers. Stress Health. (2017) 33:631–44. doi: 10.1002/smi.2750, 28371227

[46] Martinussen M Richardsen AM Burke RJ. Job demands, job resources, and burnout among police officers. J Crim Justice. (2007) 35:239–49. doi: 10.1016/j.jcrimjus.2007.03.001

[47] Eisenberger R Armeli S Rexwinkel B Lynch PD Rhoades L. Reciprocation of perceived organizational support. J Appl Psychol. (2001) 86:42–51. doi: 10.1037/0021-9010.86.1.42, 11302232

[48] Kurtessis JN Eisenberger R Ford MT Buffardi LC Stewart KA Adis CS. Perceived organizational support: a meta-analytic evaluation of organizational support theory. J Manag. (2017) 43:1854–84. doi: 10.1177/0149206315575554, 40167291

[49] Siegrist J Li J. Associations of extrinsic and intrinsic components of work stress with health: a systematic review of evidence on the effort-reward imbalance model. Int J Environ Res Public Health. (2016) 13:432. doi: 10.3390/ijerph13040432, 27104548 PMC4847094

[50] Wolfe SE Nix J. The alleged “Ferguson effect” and police willingness to engage in community partnership. Law Hum Behav. (2016) 40:1–10. doi: 10.1037/lhb0000164, 26460517

[51] Lesener T Gusy B Wolter C. The job demands-resources model: a meta-analytic review of longitudinal studies. Work Stress. (2019) 33:76–103. doi: 10.1080/02678373.2018.1529065

[52] Curelaru M Huțul TD Zamfir AL. Burnout and job satisfaction among high school teachers: a structural equation modeling approach. Teach Teach Educ. (2026) 172:105361. doi: 10.1016/j.tate.2025.105361

[53] Alegre I Mas-Machuca M Berbegal-Mirabent J. Antecedents of employee job satisfaction: do they matter? J Bus Res. (2016) 69:1390–5. doi: 10.1016/j.jbusres.2015.10.113

[54] Tian JH Kong YP Chen YW. Analysis of the current status of job burnout among human resource managers and its related factors. Manag Rev. (2011) 23:94–98. (in Chinese) doi: 10.14120/j.cnki.cn11-5057/f.2011.06.013

[55] Chuang YT Chiang HL Lin AP. Insights from the job demands-resources model: AI's dual impact on employees' work and life well-being. Int J Inf Manag. (2025) 83:102887. doi: 10.1016/j.ijinfomgt.2024.102887

[56] Jiang H. Beyond psychological costs: how negative bureaucratic encounters influence citizens' general psychological well-being. Public Adm Rev. (2026). doi: 10.1111/puar.70105

[57] Li X Seah RYT Yuen KF. Mental wellbeing in digital workplaces: the role of digital resources, technostress, and burnout. Technol Soc. (2025) 81:102844. doi: 10.1016/j.techsoc.2025.102844

[58] Bondanini G Giorgi G Ariza-Montes A Vega-Muñoz A Andreucci-Annunziata P. Technostress dark side of technology in the workplace: a scientometric analysis. Int J Environ Res Public Health. (2020) 17:8013. doi: 10.3390/ijerph17218013, 33143270 PMC7662498

[59] Afzal M Panagiotopoulos P. Coping with digital transformation in frontline public services: a study of user adaptation in policing. Gov Inf Q. (2024) 41:101977. doi: 10.1016/j.giq.2024.101977

[60] Chen J Kouhsari M. Demystifying the personal and social forces behind school digital transformation: an analysis of the job demands and resources theory through school leaders. Comput Educ. (2025) 228:105232. doi: 10.1016/j.compedu.2024.105232

[61] Wen Y Zheng F Chen L. The paradoxical logic of digital transformation of rural governance in China. E-Government. (2025) 1:113–24. (in Chinese) doi: 10.16582/j.cnki.dzzw.2025.01.010

[62] Schaufeli WB Leiter MP Maslach C Jackson SE. "The Maslach burnout inventory: general survey (MBI-GS)". In: Maslach C Jackson SE Leiter MP, editors. Maslach Burnout Inventory Manual. Palo Alto, CA: Consulting Psychologists Press (1996). 19–26.

[63] Bakker AB Demerouti E. Job demands-resources theory: taking stock and looking forward. J Occup Health Psychol. (2017) 22:273–85. doi: 10.1037/ocp0000056, 27732008

[64] Wanous JP Reichers AE Hudy MJ. Overall job satisfaction: how good are single-item measures? J Appl Psychol. (1997) 82:247–52. doi: 10.1037/0021-9010.82.2.247, 9109282

[65] Wen Y Jiang C. Attention redistribution, external resource dependence and digital village governance performance: configuration analysis based on TOE framework. Chinese Public Admin. (2023) 7:58–67. doi: 10.19735/j.issn.1006-0863.2023.07.07

[66] Li X Tsai G Yuen KF. How digital resources build capabilities for high-performing maritime project. Int J Proj Manag. (2026) 44:102814. doi: 10.1016/j.ijproman.2026.102814

[67] Brislin RW. "Translation and content analysis of oral and written material". In: Triandis HC Berry JW, editors. Handbook of Cross-Cultural Psychology: Methodology. Boston: Allyn and Bacon (1980). p. 389–444.

[68] Wu Y Liang W. The nonlinear relationship between psychological contract and burnout among Chinese government employees: a moderated mediation model. Front Public Health. (2026) 13:1729716. doi: 10.3389/fpubh.2025.1729716, 41601993 PMC12832694

[69] Hu LT Bentler PM. Cutoff criteria for fit indexes in covariance structure analysis: conventional criteria versus new alternatives. Struct Equ Model Multidiscip J. (1999) 6:1–55. doi: 10.1080/10705519909540118

[70] Cavanaugh MA Boswell WR Roehling MV Boudreau JW. An empirical examination of self-reported work stress among U.S. managers. J Appl Psychol. (2000) 85:65–74. doi: 10.1037/0021-9010.85.1.65, 10740957

[71] Thiagarajan P Chakrabarty S Taylor RD. A confirmatory factor analysis of Reilly's role overload scale. Educ Psychol Meas. (2006) 66:657–66. doi: 10.1177/0013164405282452

[72] Dorta-Afonso D Romero-Domínguez L. High-performance work systems in job demands-resources theory: implications for employee burnout and quality of life. Int J Hosp Manag. (2025) 126:104066. doi: 10.1016/j.ijhm.2024.104066

[73] Preacher KJ Rucker DD Hayes AF. Addressing moderated mediation hypotheses: theory, methods, and prescriptions. Multivariate Behav Res. (2007) 42:185–227. doi: 10.1080/00273170701341316, 26821081

[74] Fuller CM Simmering MJ Atinc G Atinc Y Babin BJ. Common methods variance detection in business research. J Bus Res. (2016) 69:3192–8. doi: 10.1016/j.jbusres.2015.12.008

[75] Benach J Vives A Amable M Vanroelen C Tarafa G Muntaner C. Precarious employment: understanding an emerging social determinant of health. Annu Rev Public Health. (2014) 35:229–53. doi: 10.1146/annurev-publhealth-032013-182500, 24641559

[76] Truelove V Oviedo-Trespalacios O. How visibility and alerts shape speed enforcement legitimacy. Transp Res Part F Traffic Psychol Behav. (2026) 119:103592. doi: 10.1016/j.trf.2026.103592

[77] Crocamo C Palpella D Cavaleri D Nasti C Piacenti S Morello P . Digital health interventions for mental health disorders: an umbrella review of meta-analyses of randomised controlled trials. Lancet Digit Health. (2025) 7:100878. doi: 10.1016/j.landig.2025.100878, 40610362

[78] Gillet N Morin AJS Huart I Odry D Chevalier S Coillot H . Self-determination trajectories during police officers' vocational training program: a growth mixture analysis. J Vocat Behav. (2018) 109:27–43. doi: 10.1016/j.jvb.2018.09.005

[79] Zhou W Gu Y Peng J. The effect of government auditors' work stress on job burnout: the roles of psychological contract and leader-member exchange. Manag Rev. (2020) 32:229–38. doi: 10.14120/j.cnki.cn11-5057/f.2020.09.019

[80] Zhang K Lu G Wang J. A path model of the role of psychological capital in the relationship between job stress and job burnout. Stud Psychol Behav. (2014) 12:91–6.

