# Supplementary material for: Regulation, resources, and job burnout among traffic auxiliary police in China: evidence from G City
Source: Front Public Health. 2026 Aug 4;14:1855841. doi: 10.3389/fpubh.2026.1855841 (PMC13482916; doi:10.3389/fpubh.2026.1855841)
Supplement: Supplementary file 1 [file Supplementary_file_1.docx]

**Appendix 1.** Scale items

| **Variables** | | **Survey question** | **Coding rule** |
| --- | --- | --- | --- |
| **Construct** | **Item** |  |  |
| Dependent variable | | | |
| burnout | EE | 1 Work makes me feel physically and mentally exhausted | 1 = almost never, 2 = occasionally, 3 = sometimes, 4 = often, 5 = almost daily |
|  |  | 2 I feel tired when I wake up and think about going to work |  |
|  |  | 3 After a full day of work, I feel completely exhausted |  |
|  |  | 4 My work consumes a great deal of my emotional energy |  |
|  |  | 5 My work makes me feel highly stressed |  |
|  | DP | 6 I have started to question the meaning of my work |  |
|  |  | 7 I have become accustomed to doing my work perfunctorily, just to complete tasks |  |
|  |  | 8 I am becoming increasingly indifferent toward my colleagues |  |
|  | DA | 9 I am able to make use of my professional expertise in my job |  |
|  |  | 10 I can effectively solve work-related problems |  |
|  |  | 11 I can calmly manage my work-related emotions |  |
|  |  | 12 I feel that my work is helpful to others |  |
| Independent variables | | | |
| JRD | RPC | 13 The workload required by organizational rules is excessive | 1 = strongly disagree, 2 = disagree, 3 = neutral, 4 = agree, 5 = strongly agree |
|  |  | 14 Organizational regulations generate many emergent tasks or assignments, creating substantial pressure for me |  |
|  |  | 15 Multiple work rules force me to deal with burdensome problems |  |
|  | RRT | 16 Shifts in my work role expose me to conflicting institutional demands within the organization |  |
|  |  | 17 I feel unclear about the unit’s responsibility system and my work role |  |
|  |  | 18 Overtime work arrangements often make me feel overstretched |  |
|  | RED | 19 A complex institutional work environment affects my ability to fulfill family responsibilities normally |  |
|  |  | 20 Different work environments and institutional arrangements frequently force me to change planned non-work activities |  |
| JR | ORS | 21 My work performance is recognized by the organization |  |
|  |  | 22 My occupational needs receive attention from the organization |  |
|  |  | 23 My work receives human, material, informational, and emotional support |  |
|  | CPF | 24 The compensation mechanism matches performance expectations |  |
|  |  | 25 The promotion system supports my career development plans |  |
|  |  | 26 My efforts and achievements receive reasonable returns through reward and performance evaluation mechanisms |  |
|  | WAR | 27 I can independently choose how to complete work tasks |  |
|  |  | 28 My work schedule is flexible and controllable |  |
|  |  | 29 I have discretionary space in handling work matters |  |
| Mediating variable | | | |
| JS | support | 30 I often receive care and support at work | 1 = strongly disagree, 2 = disagree, 3 = neutral, 4 = agree, 5 = strongly agree |
|  | belonging | 31 I feel attached to the organization and am willing to stay for the long term |  |
|  | overall satisfaction | 32 Overall, I am very satisfied with my current job |  |
| Moderating variable | | | |
| DR | perceived usefulness | 33 Digital technology can improve work efficiency | 1 = strongly disagree, 2 = disagree, 3 = neutral, 4 = agree, 5 = strongly agree |
|  | use frequency | 34 I use intelligent tools in my daily work |  |
| Control variables | | | |
| gender | | 1 = Female, 2 = Male | |
| age | | 1 = 18-30, 2 = 30-40, 3 = 40-50, 4 = 50-60, 5 = over 60 | |
| education | | 1 = Primary school and below, 2 = Junior high school, 3 = Senior high school/technical secondary school, 4 = College/Bachelor's degree, 5 = Postgraduate degree | |
| rank | | 1 = Low, 2 = Relatively low, 3 = Moderate, 4 = Relatively high, 5 = High | |

*Note. (1) EE = emotional exhaustion; DP = depersonalization; DA = reduced personal accomplishment (reverse-coded); RPC = regulatory pressure constraints; RRT = regulatory role tension; RED = regulatory environment discrepancies; ORS = organizational resource support; CPF = career performance feedback; WAR = work autonomy resources; JS = job satisfaction; DR = digital resources. (2) EE, DP, and DA are the three dimensions of job burnout; RPC, RRT, and RED are the three dimensions of job regulation demands (JRD); ORS, CPF, and WAR are the three dimensions of job resources (JR).*

**Appendix 2.** Cronbach's α, CR, and AVE of the main variables

| **Variables** | **Cronbach’s α** | **CR** | **AVE** |
| --- | --- | --- | --- |
| burnout | 0.942 | 0.945 | 0.543 |
| EE | 0.948 | 0.951 | 0.827 |
| DP | 0.904 | 0.907 | 0.769 |
| DA | 0.905 | 0.908 | 0.712 |
| JRD | 0.923 | 0.926 | 0.589 |
| RPC | 0.936 | 0.938 | 0.835 |
| RRT | 0.891 | 0.895 | 0.74 |
| RED | 0.874 | 0.878 | 0.782 |
| JR | 0.918 | 0.921 | 0.564 |
| ORS | 0.885 | 0.889 | 0.73 |
| CPF | 0.852 | 0.857 | 0.667 |
| WAR | 0.817 | 0.824 | 0.512 |
| JS | 0.831 | 0.836 | 0.63 |
| DR | 0.865 | 0.87 | 0.763 |

*Note. Burnout = job burnout; EE = emotional exhaustion; DP = depersonalization; DA = reduced personal accomplishment; JRD = job regulation demands; RPC = regulatory pressure constraints; RRT = regulatory role tension; RED = regulatory environment discrepancies; JR = job resources; ORS = organizational resource support; CPF = career performance feedback; WAR = work autonomy resources; JS = job satisfaction; DR = digital resources.*

**Appendix 3.**  HTMT (heterotrait-monotrait ratio) values for the core latent constructs

| Variables | burnout | JRD | JR | JS | DR |
| --- | --- | --- | --- | --- | --- |
| burnout | - |  |  |  |  |
| JRD | 0.72 | - |  |  |  |
| JR | 0.68 | 0.55 | - |  |  |
| JS | 0.75 | 0.62 | 0.71 | - |  |
| DR | 0.48 | 0.41 | 0.53 | 0.57 | - |

*Note. (1) HTMT values are reported for the core latent constructs included in the discriminant-validity assessment, and all values are < 0.85. (2) Burnout = job burnout; JRD = job regulation demands; JR = job resources; JS = job satisfaction; DR = digital resources.*
